# Cuminaldehyde Effects in a MIA-Induced Experimental Model Osteoarthritis in Rat Knees

**DOI:** 10.3390/metabo13030397

**Published:** 2023-03-08

**Authors:** Sebastião Vieira de Morais, Priscylla Gouveia Mendonça, Cleydlenne Costa Vasconcelos, Paloma Larissa Arruda Lopes, João Batista Santos Garcia, Natalia Tabosa Machado Calzerra, Thyago Moreira de Queiroz, Silvia Tereza de Jesus Rodrigues Moreira Lima, Gyl Eanes Barros Silva, Alberto Jorge Oliveira Lopes, Maria do Socorro de Sousa Cartágenes, Gerson Ricardo de Souza Domingues

**Affiliations:** 1 Biological and Health Sciences Center, Federal University of Maranhão, Av. dos Portugueses 1966, São Luís 65085-580, MA, Brazil; 2 Department of Physiology and Pharmacology, Federal University of Pernambuco, Av. Prof. Moraes Rego, 1235—Cidade Universitária, Recife 50670-901, PE, Brazil; 3 Laboratory of Nutrition, Physical Activity and Phenotypic Plasticity, Federal University of Pernambuco, Av. Prof. Moraes Rego, 1235—Cidade Universitária, Recife 50670-901, PE, Brazil; 4 Hospital Universitário Presidente Dutra, HUPD, Federal University of Maranhão, R. Barão de Itapari, 227—Centro, São Luís 65020-070, MA, Brazil; 5 Federal Institute of Science Education and Technology of Maranhão—Campus Monte Castelo, Chemistry Postgraduate Program, Av. Getúlio Vargas, 04—Monte Castelo, São Luís 65030-005, MA, Brazil; 6 State University of Rio de Janeiro School of Medicine, Av. Prof. Manoel de Abreu, 444, Vila Isabel—Rio de Janeiro 20550-170, RJ, Brazil

**Keywords:** new drugs agents, natural products, medicinal chemistry, biological activity

## Abstract

Osteoarthritis (OA) is a chronic degenerative disease that has a significant global impact. It is associated with aging and characterized by widespread joint destruction. Cuminaldehyde is a biologically active component of essential oils that has shown promise in the treatment of nociceptive and inflammatory diseases. This study investigated the effects of cuminaldehyde on an experimental model of osteoarthritis induced in rat knees. Cuminaldehyde was found to be as effective as indomethacin in reducing pain in all evaluated tests, including forced walking, functional disability of weight distribution on the legs, and spontaneous pain in animals with osteoarthritis. The knees of animals treated with cuminaldehyde had significantly higher radiographic and histopathological scores than those of animals that did not receive the treatment. Cuminaldehyde also modulated the production of pro-inflammatory cytokines. In vitro assays showed that cuminaldehyde preferentially inhibits COX-2 enzyme activity. In silico studies demonstrated that cuminaldehyde has satisfactory energy affinity parameters with opioid receptors and COX-2. These findings suggest that cuminaldehyde’s anti-inflammatory activity is multifactorial, acting through multiple pathways. Its nociceptive activity occurs via central and peripheral mechanisms. Cuminaldehyde modulates the immune response of the inflammatory process and may be considered a leading compound for the development of new anti-inflammatory and analgesic drugs.

## 1. Introduction

Osteoarthritis (OA) is the most common joint disease worldwide, affecting about 10% of men and 18% of women over the age of 60. Due to its progressive nature, it is irreversible in most cases, resulting in severe and recurrent inflammatory and painful symptoms, with a reduction or total loss of functional capacity of the affected joint. OA also leads to loss of productivity due to time off work and produces high financial costs for health services, mainly related to pain control and arthroplasties. These costs can range between 10% and 25% of the gross domestic product [1,2,3,4,5].

OA is a multifactorial disease [6], that results from a complex interaction of genetic, metabolic, biochemical, and biomechanical factors, which then activate the inflammatory response, leading to chronic degeneration of cartilage, changes in the subchondral bone, and synovial tissue [7]. It is also classified as an inflammatory disease, characterized by an increase in pro-inflammatory cytokines such as interleukin 1β (IL1β) and tumor necrosis factor alpha (TNF-α). These cytokines decrease collagen synthesis and promote an increase in catabolic mediators, such as metalloproteinases, which degrade components of the extracellular matrix and subchondral bone [8,9].

The articular damage caused by OA affects not only the joint, but also all its components, including ligaments, capsule, synovial membrane, and periarticular muscles. It results from phenotypic alterations in chondrocytes, which are fully differentiated cells responsible for the function of adult hyaline articular cartilage. In OA, articular chondrocytes respond to the accumulation of biochemical and biomechanical harmful insults, changing to a state of hypertrophy and degradation. This leads to anatomical, biochemical, molecular, and biomechanical alterations, resulting in articular cartilage damage, remodeling, and sclerosis of the subchondral bone, with the formation of marginal osteophytes [7,10].

During hypertrophic growth, a transitional stage of chondrocyte development occurs in the growth plate chondrocytes that culminates in bone formation. However, this hypertrophy of the chondrocytes can be catastrophic as it initiates and perpetuates a cascade of events that ultimately lead to permanent cartilage damage [11].

The available treatments for OA mainly rely on the use of analgesics and non-steroidal anti-inflammatory drugs (NSAIDs), which are inhibitors of the enzyme cyclo-oxygenase-2 (COX-2). However, the use of these drugs is still insufficient as they cannot reverse the progression of the disease and present significant side effects. Moreover, the pain is often refractory to current treatments [12,13], due to prolonged use of NSAIDs, and, despite providing temporary relief, increases the risk of renal dysfunction, gastrointestinal complications, and cardiovascular diseases [14].

Given the high prevalence of pain and the complications associated with its treatment, there is a growing need to develop new analgesic medications that can improve both efficacy and safety. Traditional medicine has long utilized various herbal remedies for pain control, and, as such, recent studies have focused on medicinal plants and their compounds as a potentially important source for drug discovery due to their beneficial effects on pain management [15].

Cuminaldehyde, also known as 4-isopropylbenzaldehyde, is a benzaldehyde compound with a substituted isopropyl group in position 4. It is the major component of cumin (*Cuminum cyminum* L.) and is also present in the essential oils of other plants such as *Carum carvi* L., *Cinnamomum cassia* (L.) J. Presl, and *Cinnamomum verum* J. Presl. [16], which exhibits several biological activities, including antinociceptive and anti-neuropathic effects upon stimulation of opioid receptors, L-arginine/NO/cGMP, and suppression of inflammatory cytokines [17]. It also has an anti-inflammatory effect by inhibiting NF-κB and mitogen-activated protein kinases ERK and JNK signaling pathways [18].

Although some studies suggest that cuminaldehyde has anti-inflammatory and analgesic properties, which may be beneficial for reducing pain and inflammation associated with osteoarthritis [16,17,18], more research is needed to determine its effectiveness as a treatment for osteoarthritis. Thus, the present study evaluated the activity of cuminaldehyde on clinical, immunological, radiological, and histological parameters in animals affected with OA.

## 2. Materials and Methods

### 2.1. Cuminaldehyde

The reagent used in the present study was obtained commercially (#135178 Sigma-Aldrich; San Luis, MO, USA), with a purity of 98%, and stored at room temperature until the moment of its use.

### 2.2. Animals and Ethics Aspects

The in vivo experimental model used in this study consisted of adult male Wistar rats (*Rattus norvegicus*) aged between 27 and 31 days, with weights ranging from 280 to 300 g, obtained from UFMA’s Central Vivarium (Biotério Central). Throughout the entire experimental period, the rats were fed standard chow and water ad libitum and were kept under controlled conditions of temperature (23 ± 1 °C), humidity (40–60%), and a 12-h light–dark cycle.

All in vivo experimental assessment protocols were approved and authorized by the Federal University of Maranhão (UFMA) Ethics Committee in Animal Use (ECAU) on 3 December 2019, under protocol code 23115.031386/2019-28. The experiments were conducted according to the International Association for the Study of Pain (IASP) Guidelines for the Use of Animals in Research.

### 2.3. Experimental Design

The animals were divided into groups (N = 6) at random. The SHAM group did not undergo any intervention, while the other groups received an intra-articular injection of sodium monoiodoacetate (MIA) (2 mg in 25 µL) to induce osteoarthritis in the right knee, followed by their respective treatments: saline solution (NaCl 0.9%) at a dose of 1 mL/kg/day (vehicle) (CTL−), Indomethacin^®^ at a dose of 2.5 mg/kg/day (CTL+), and cuminaldehyde at a dose of 50 mg/kg/day. The animals were given the daily dose orally via the orogastric route using the gavage method from day 3 to day 28. These groups were assessed for antinociceptive activity by incapacitation (Weight Bearing test) and motor activity (Rotarod test) every 7 days and were euthanized on the 28th day after OA induction using an intraperitoneal solution of ketamine hydrochloride (300 mg/kg) and xylazine hydrochloride (30 mg/kg). The right paw with OA-induced was collected for radiographic and histopathological analyses.

### 2.4. In Vivo Clinical Assessments

#### 2.4.1. Evaluation of Motor Activity/Forced Deambulation (Rotarod Test)

During the forced walking test, the animals were placed on a rotating bar (IITC model, Life Science) that rotated at 16 rpm for 300 s. To assess the use of the affected limb, the animals were observed and scored on a scale from 1 to 5, where 5 represented normal paw use, 4 represented mild claudication, 3 represented severe claudication, 2 represented intermittent disuse of the affected paw, and 1 represented complete disuse of the affected paw [19].

#### 2.4.2. Incapacitation/Weight Distribution Test on Hind Legs (Weight Bearing)

To assess the use of the affected limb, the animals were placed in an angled glass chamber with each hind leg resting on different platforms. Weight exerted on each hind leg was measured in grams for five seconds and the final measurement was an average of three [20]. Weight distribution changes were calculated using the following formula:

Weight distribution (%) = APW∕(APW + CPW) × 100(1)

where APW represents the weight of the affected paw and CPW represents the weight of the contralateral paw.

#### 2.4.3. Mouse Grimace Scale (MGS)

The Mouse Grimace Scale (MGS) is a reliable method used to assess spontaneous pain in laboratory animals by analyzing changes in facial expressions. To discern the subjective sensation of facial pain, the criteria for evaluation were adapted from Sotocina et al. [21]. A score of “0” indicates no pain, “1” indicates moderate pain, and “2” indicates severe pain. The facial pain was evaluated by observing changes in the eyes, nose/cheek protuberance, ears, and moustache.

### 2.5. Radiographical Analysis

The radiographs of the lower limbs of the animals were obtained using an X-ray machine with radiological protection and model specifications as follows: PHILIPS Medical Systems Ltd.a, Rotax sealing unit, KVP 125, #Series PA-DXJL.10.001, model KL.90.30/50. The images were taken on day 28 after the animals had been euthanized and only the limb was used for X-ray. They were positioned on the chassis at a distance of 115 cm from the focus. The developer used was the DIRECT DIGITIZER, Model DRYPRO SIGMA S/N 002650, P/N 9G9001, REF A4A9.

### 2.6. Histopathological Analysis

Knee joints were harvested and fixed in 10% (*v*/*v*) formalin for 24 h. Next, they were demineralized in a 10% formic acid solution at pH 4.5, under moderate vacuum for 10 days. The joints were then washed with running water, dehydrated in increasing alcohol solutions, diaphanized in xylol, and embedded in paraffin blocks. A paraffin block inclusion protocol was performed, followed by specific staining of the proteoglycans of the organic cartilage matrix using 0.5% O-safranin. To serve as a histological control of the cartilage, Toluidine Blue staining was also performed. This staining specifically stains the proteoglycans of the organic matrix of cartilage. Various parameters such as the inflammatory exudate, involvement of inflammatory exudate (perivascular, interstitial, and synovial epithelium), fibrinoid deposits, and necrosis were subjectively interpreted based on the findings of previous authors who utilized experimental models of osteoarthritis in Wistar rats. The samples were sectioned to a thickness of 4 μm [22].

The study utilized the Osteoarthritis Research Society International (OARSI) score, which is a semi-quantitative method for evaluating the extent and severity of osteoarthritic lesions in cartilage. The OARSI histopathological assessment system for cartilage with OA is based on the histological features of OA progression, and the grading system is defined by the depth of OA progression within the cartilage. This grading system is used to assess the severity and biological progression of the osteoarthritic process. The most severe injury observed on the slide is classified as the overall grade, regardless of the extent of the injury. The grading system ranges from 0 to 6, with 0 indicating a morphologically intact cartilage, grade 1 indicating an intact surface with possible focal lesions or abrasion, grade 2 indicating discontinuity on the joint surface, grade 3 comprising vertical cracks, grade 4 indicating erosions, grade 5 consisting of bone denudations, sclerotic or fibro-cartilaginous tissue repair or both, and grade 6 indicating bone remodelling and deformation with articular surface contour changes [23].

### 2.7. Cytokines Evaluations

The enzyme-linked immunosorbent assay (ELISA) was employed to quantify cytokines (IFN-γ, IL-6, and IL-10) in blood serum samples collected from mice at the time of euthanasia, following the technical instructions of the cytokine dosing kits and equipment. The ELISA kit used in this study was purchased from R&D Systems^®^ (Minneapolis, MN, USA).

### 2.8. In Vitro Activity on Cyclooxygenase

The experiment followed the manufacturer’s protocol for the Cox Colorimetric Inhibitor Screening assay (Cayman Chemical^®^, Ann Arbor, MI, USA). A 96-well plate was used, and inhibition tests were carried out in triplicate for each of the three concentrations tested (2, 10, and 50 µg/mL) of cuminaldehyde, as well as for the reaction controls. The three wells labelled “BW” were treated with 160 µL of Tris-HCl buffer, 10 µL of Heme, and 10 µL of a 70% ethanol solvent used to dilute the samples. The three wells labelled “A” received 150 µL of Tris-HCl buffer, 10 µL of Heme, 10 µL of COX enzyme (COX-1 or COX-2), and 10 µL of 70% ethanol solvent. The wells with cuminaldehyde were treated with 150 µL of Tris-HCl buffer, 10 µL of Heme, 10 µL of COX enzyme (COX-1 or COX-2), and 10 µL of a cuminaldehyde sample diluted with a 70% ethanol solvent at concentrations of 2, 10, and 50 µg/mL. After adding all the reagents to each well, the plate was carefully shaken for a few seconds and incubated for 5 min. Next, 20 µL of a solution containing the colorimetric substrate was added to each well, followed by the addition of 20 µL of arachidonic acid, the substrate of the enzymatic reaction catalysed by COX. The plate was shaken again and incubated for 2 min at 25 °C, after which the absorbance was measured at 590 nm.

### 2.9. In Silico Assay

#### 2.9.1. Structures of the Compounds

Cuminaldehyde (C_10_H_12_O) and the other drugs were structurally schematized in three dimensions (3D) with the software GaussView 6.1 (Semichem Inc., Shawnee Mission, KS, USA) [24] and had their geometric and vibrational properties calculated (optimization) in vacuum at the density functional theory (DFT) method level using the hybrid functional B3LYP combined with the base 6-31 ++ G (d, p) with the software Gaussian 16 [25] to obtain the atomic and molecular electronic properties that correlate with the possible biological activity.

#### 2.9.2. Target Structures

The structures of COX-2 (id #1DDX), the kappa (κOR—id #6B73), mu (µOR—id #6DDF) and delta (δOR—6PT3) type opioid receptors (OpR) obtained through experimental techniques (X-ray crystallography, electron microscopy, nuclear magnetic resonance) from the Protein Data Bank (PDB) were utilized.

#### 2.9.3. Molecular Docking

The 3D structures of COX-2 (id #1DDX) and opioid receptors (κOR—id #6B73, µOR—id #6DDF, and δOR—6PT3) obtained from the Protein Data Bank (PDB) were used for molecular docking analysis. The AutoDock Vina package [26] was used for docking, and the AutoDock Tools 1.5.7 module was used to prepare and analyse the computational calculations. The structures of cuminaldehyde or drugs were optimized and positioned in the central portion of the catalytic site of the selected COX-2 (Arg120) and OpR (Asp128 for OpR-delta, Asp138 for OpR-kappa, and Asp147 for OpR-mu).

The target structures were prepared by adding Gasteiger charges and polar hydrogens, and water molecules, drugs, and artifacts were removed [27]. The ligands were kept free and mobile, while the target structures were kept rigid. The best ‘ligand + receptor’ complexes were selected based on binding free energy and inhibition constants by visual inspection and residue analyses that showed the best interaction with the ligand [28]. The molecular analyses and complex representations were obtained using the UCSF Chimera package and PoseView [29,30].

### 2.10. Statistical Analysis

Statistical analysis was performed to compare the mean values of different experimental groups using Student’s t-test or bivariate analysis of variance (two-way ANOVA), followed by Tukey’s test. Two-way ANOVA was used to evaluate two sources of variability. Statistical significance was defined as a *p*-value less than 0.05. GraphPad Prism^®^ software (version 7.0, GraphPad Software, San Diego, CA, USA) was used for data analysis.

## 3. Results

### 3.1. Motor Activity/Forced Deambulation (Rotarod Test) Evaluation

**Figure 1:**
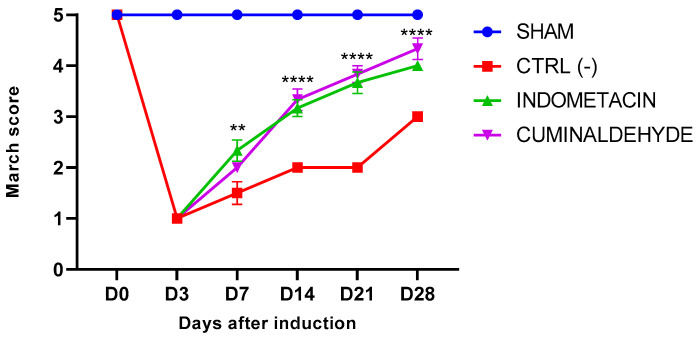
Effects of cuminaldehyde on the motor activity/forced ambulation of rats with induced OA, evaluated using the Rotarod test score. The animals were administered oral saline (CTL−), indometacin (CTL+), and cuminaldehyde (50 mg/kg) from D3 to D28, and were evaluated on days 7, 14, 21, and 28, after OA induction. Data are represented as means ± standard error of the means (SEM). The SHAM group represents animals without osteoarthritis and without any treatment. The statistical analysis was performed using two-way ANOVA followed by Tukey’s test. Significant differences were denoted by ** at *p* < 0.001 and **** at *p* < 0.0001. (D = day).

The deambulation score of all groups induced with OA was similarly reduced in D3 (Figure 1), demonstrating effective induction. Treatment with cuminaldehyde showed a significant difference (*p* < 0.0001) compared to treatment with saline, as evidenced by a progressive improvement in the gait parameter compared to the negative control group. The improvement was statistically equal to the treatment with indomethacin, from the seventh day after induction of OA throughout the experimental period (Figure 1).

### 3.2. Incapacitation/Weight Distribution Test on Hind Legs (Weight Bearing)

**Figure 2:**
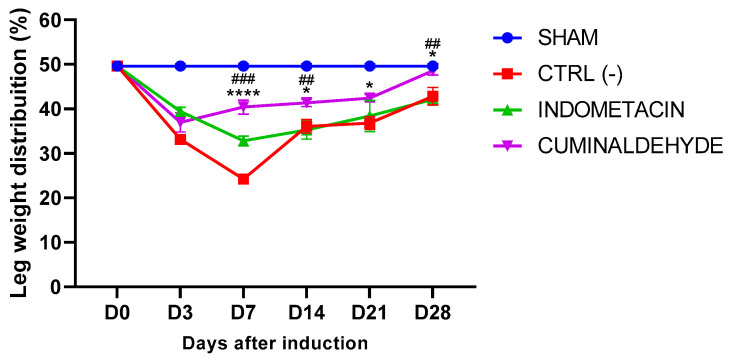
Effects of cuminaldehyde on the extent of disability in rats with induced osteoarthritis (OA), evaluated using the weight bearing test. The results are expressed as means ± standard error of the means (SEM). The healthy group (SHAM) represents animals without osteoarthritis or any treatment. The statistical analysis, conducted using two-way ANOVA followed by Tukey’s test, revealed significant differences (*p* < 0.05) between cuminaldehyde-treated animals and both the saline group (CTRL-) and the indomethacin (positive control) group at D7, D14, and D28. The symbol * indicates significant differences compared to the saline group, and the symbol # indicates significant differences compared to the indomethacin group. Furthermore, the symbol **** represents *p* < 0.0001, ## represents *p* < 0.01, and ### represents *p* < 0.001. (D = day).

The SHAM group, which did not receive any treatment, demonstrated symmetrical support on both hind limbs at the beginning and end of the experiment, with a score of approximately 50% indicating the absence of joint pain. On the other hand, at days 7, 14, and 28, animals treated with cuminaldehyde showed significant improvements (*p* < 0.05) in weight distribution on both legs compared to the saline and indomethacin groups. At day 28, the weight distribution on the legs of cuminaldehyde-treated animals was normalized (Figure 2).

### 3.3. Rating by Mouse Grimace Scale

**Figure 3:**
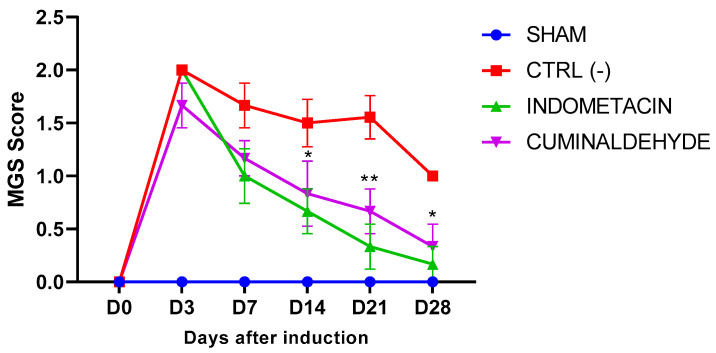
Effect of cuminaldehyde in the evaluation of spontaneous pain by the Mouse Grimace Scale score. Results are expressed as mean ± standard error of the means (SEM). * Significant differences, at *p* < 0.05; ** at *p* < 0.01. * compared to saline (CTRL-) group. (Two-way ANOVA; Tukey). (D = day).

Following the induction of OA, all groups demonstrated a similar increase in spontaneous pain, as evidenced by the MGS. However, from day 14 onwards, animals treated with cuminaldehyde showed a significant improvement (*p* < 0.05) in pain scores compared to the CTRL (-) group. Furthermore, cuminaldehyde demonstrated analgesic activity comparable to indomethacin throughout the experimental period (Figure 3).

### 3.4. Radiographic Analysis

**Figure 4:**
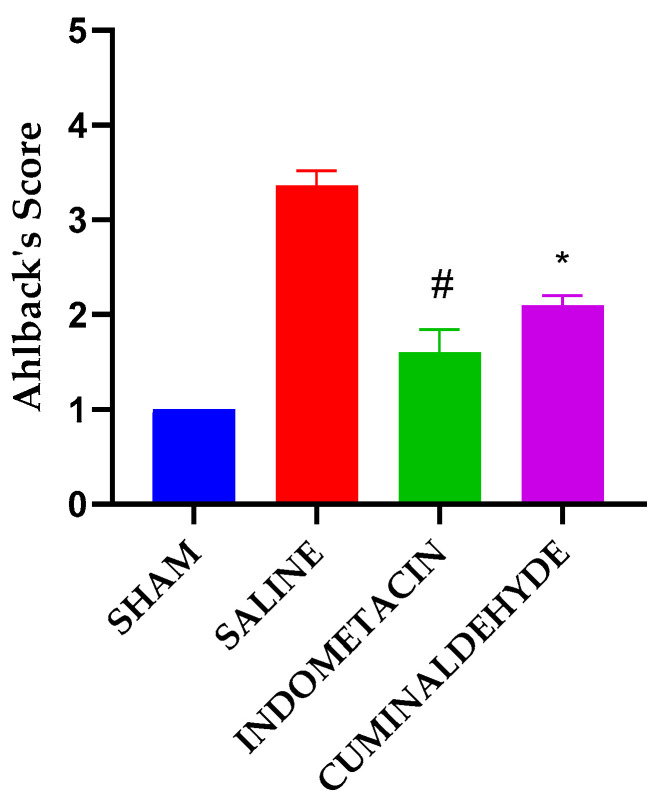
Degree of joint involvement in rats treated with cuminaldehyde, following the Alhback’s Score Data are represented as means ± standard error of the means (SEM). This analysis was performed with the knees collected on day 29 after OA induction. * Significant differences at *p* < 0.05 compared to CTRL (-) group (saline); # Significant differences at *p* < 0.05 compared to cuminaldehyde group. (one-way ANOVA; Dunnett).

Radiographic examinations of the rats’ knees showed differences in the Ahlback Score, indicating the degree of joint damage. The OA-induced rats exhibited significant joint space loss. Compared to the negative control group, both the indomethacin and cuminaldehyde treatment groups showed a statistically significant difference, with a lower score indicating less bone loss, 7 mm along the external or internal joint margins from a line drawn perpendicular to the tibial axis and tangent to the unaffected joint surface. However, Sidak’s statistical test was used to confirm the significance of the difference (Figure 4).

### 3.5. Histopathological Analysis

**Figure 5:**
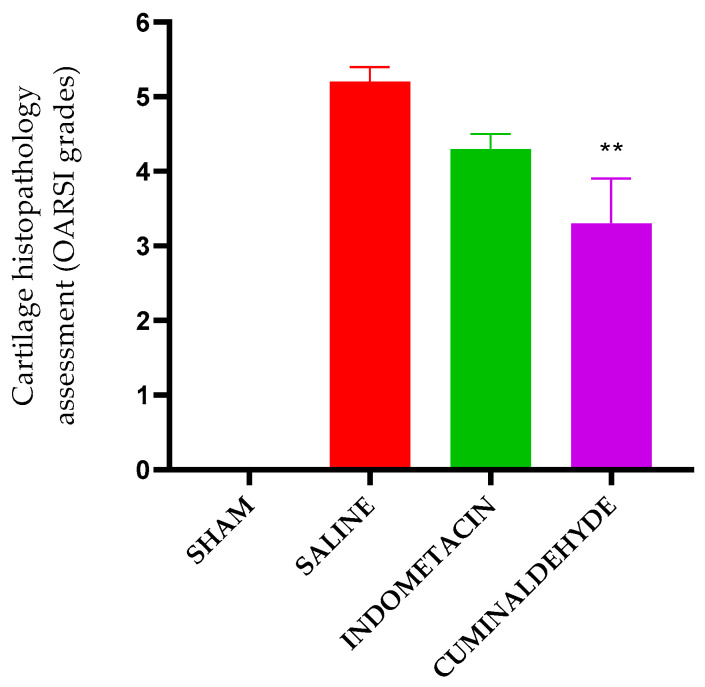
Results of histopathological cartilage evaluations, classified by the Osteoarthritis Research Society International (OARSI) scoring system. The analysis was performed on the knees collected on the 29th day after OA induction. The groups included the untreated and OA uninduced (SHAM), the induced OA and untreated group (CTL−), the induced OA and Indomethacin-treated group, and the induced OA and Cuminaldehyde-treated group. The *Y*-axis represents the histopathological evaluation of cartilage according to the OARSI histological classification system: Grade 0—surface intact and cartilage intact; Grade 1—surface intact; Grade 2—surface discontinuity; Grade 3—vertical fissures; Grade 4—erosion; Grade 5—denudation; and Grade 6—deformation). ** Significant differences, at *p* < 0.005 compared to the saline group (CTL−). (one-way ANOVA; Tukey).

**Figure 6:**
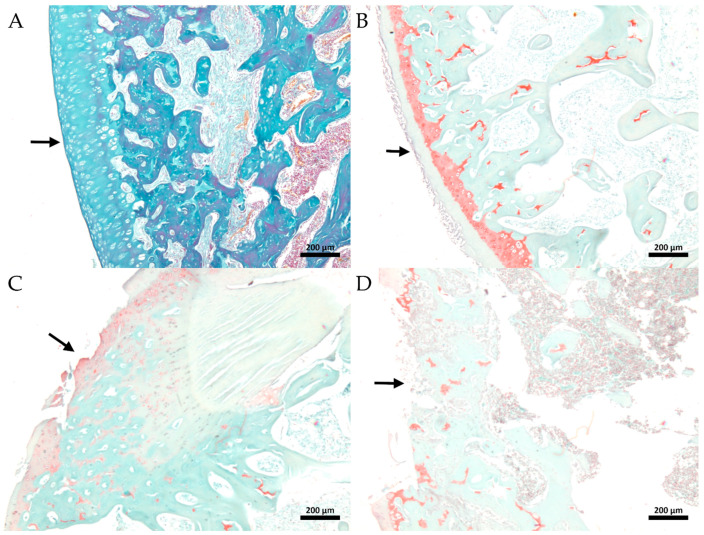
Summary of the Osteoarthritis Research Society International (OARSI) cartilage histopathology evaluation systems in different animal groups. Panel (**A**) depicts the sham group with normal cartilage width, classified as grade 0. Panel (**B**) shows an animal from the cuminaldehyde group with moderate degeneration, classified as grade 3. Panel (**C**) shows an animal from the indometacin group with erosion to the calcified cartilage extending to approximately 75% of the articular surface, classified as grade 4/5. Panel (**D**) shows an animal from the saline group with processes of microfracture, repair, and bone remodeling, classified as grade 5/6. The cartilage matrix was stained with Safranin-O, and the original magnification was 50x. The bar represents 200 µm. The arrow indicates the surface face of the articular cartilage.

The severity or biological progression of the osteoarthritic process was classified according to the Osteoarthritis Research Society International (OARSI) score. Animals treated with cuminaldehyde showed statistical significance in OA progression with an average score of 3.3 (±0.6), which was significantly lower than the saline control group (CTL-) with a mean score of 5.2 (±0.2). The indometacin group had a mean score of 4.3 (±0.2) (Figure 5 and Figure 6). Thus, the animals treated with cuminaldehyde exhibited a better histological profile of knee cartilage, with a lower OARSI score classification than the saline group.

### 3.6. Cytokine Analysis

**Figure 7:**
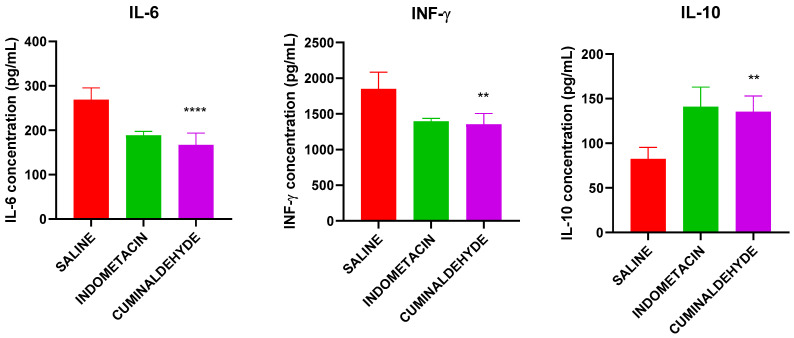
Concentration of the cytokines IL-6 (A), INF-y (B) and IL-10 (C) and TNF-a (D), evaluated by ELISA of the serum of the animals used in the experiments. **, **** Significant differences, at *p* < 0.005 and 0.0001, respectively, compared to the saline group (CTL−); (one-way ANOVA; Tukey).

The concentration of IL-6 and INF-γ in animals treated with cuminaldehyde was significantly lower than that in animals in the saline group (*p* < 0.0001 and *p* < 0.001, respectively). No significant differences were observed in these cytokines between animals in the cuminaldehyde and indomethacin groups. The concentration of IL-10 was significantly increased in animals treated with cuminaldehyde compared to the saline group (*p* < 0.001) (Figure 7).

### 3.7. Inhibition of Cyclooxygenase 1 and 2

**Figure 8:**
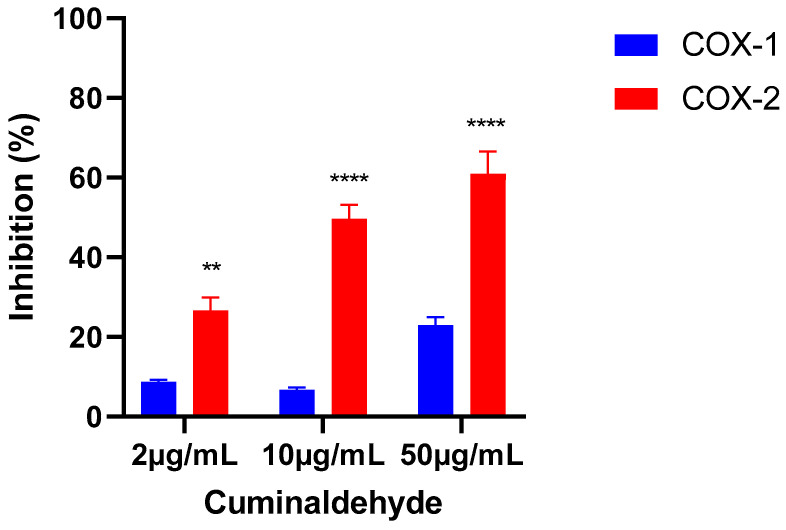
The percentual in vitro inhibition of cyclooxygenase 1 and 2 (COX-1 and COX-2) produced by cuminaldehyde at three tested concentrations: 2 µg/mL, 10 µg/mL, and 50 µg/mL. The asterisks indicate significant differences, with ** representing *p* < 0.005 and **** representing *p* < 0.0001 when comparing the inhibition of COX-2 with COX-1. The statistical analysis was performed using two-way ANOVA with Sidak’s multiple comparison test.

The results of the cyclooxygenase 1 and 2 inhibition tests showed that cuminaldehyde had low inhibition for COX-1 at all three concentrations tested, with the highest inhibition observed at the highest concentration (50 µg/mL). In contrast, cuminaldehyde showed more pronounced inhibition for COX-2, inhibiting 60.9% of this isoform’s activity at a concentration of 50 µg/mL. These results suggest that cuminaldehyde exhibits greater selectivity for COX-2 over COX-1, with statistically significant differences in inhibition observed between the two isoforms (*p* < 0.0001) (Figure 8).

### 3.8. Molecular Docking

Cuminaldehyde showed significant electronic affinity parameters with COX-2 (−7.2 kcal/mol) and all types of opioid receptors (OR) evaluated, with kappa (−6.80 kcal/mol), delta (−6.70 kcal/mol), and mu (−6.00 kcal/mol) types standing out. To validate the employed protocol, redocking of CVV, Morphine, and Naltrindole in the OpR kappa, mu, and delta, respectively, was performed. The root mean square deviation (RMSD) between predicted redocking conformations and the observed X-ray crystal structure was always less than 1.54 Å for all drugs, indicating a valid docking protocol that successfully predicted the spatial conformation of the ligand in the target’s active site. During redocking, it was found that all drugs had higher energy affinity parameters than cuminaldehyde, as shown in Table 1.

**Figure 9:**
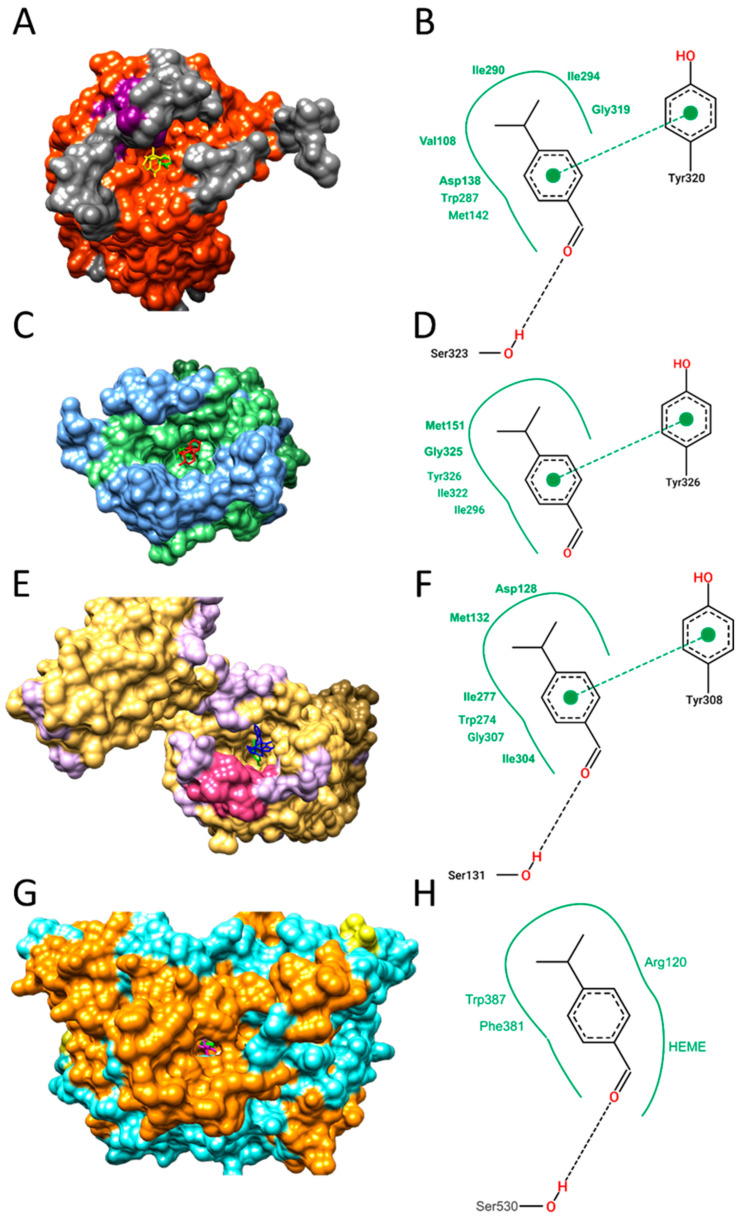
The spatial conformation of kappa, mu, and delta ORs (PDB IDs 6B73, 6DDF, and 6PT3, respectively) and COX-2 interacting with cuminaldehyde (shown in green) is illustrated in panels (**A**,**C**,**E**,**G**), respectively. Panels (**B**,**D**,**F**,**H**) show the two-dimensional diagram of the contacts made by cuminaldehyde with the amino acid residues of the active site of kappa, mu, and delta ORs, and COX-2, respectively, obtained by molecular docking. Traditional opioid drugs, such as CVV (shown in yellow in Figure 9A), morphine (in red in Figure 9C), and naltrindole (in blue in Figure 9E), as well as indometacin (in magenta in Figure 9G) are also depicted. Hydrogen bonds are represented by dashed black lines, van der Waals interactions by full green lines, and π-π interactions by dashed green lines.

Furthermore, the molecular docking results indicated that cuminaldehyde had favorable binding free energy parameters, and it was predicted to bind to the same region as traditional opioid drugs, as shown in Figure 9. This suggests that cuminaldehyde may have potential as an opioid receptor agonist and COX-2 inhibitor.

## 4. Discussion

The rat knee model induced by intra-articular monosodium iodoacetate is a valuable tool for research related to osteoarthritis, as it produces significant changes in joint movements, tactile allodynia, progressive radiological degeneration, and microscopic inflammation of the synovial membrane. This model causes chondrocyte degeneration by inhibiting 3-phosphate-glyceraldehyde, which serves as a marker for evaluating osteoarthritis [31,32].

In osteoarthritis (OA), the pathogenesis involves the action of inflammatory neuromodulators on nociceptor terminals, leading to peripheral sensitization. This results in allodynia and hyperalgesia, which are characterized by a decreased pain threshold, induction of ectopic discharges, and increased sodium channels [33,34].

Cuminaldehyde is a naturally occurring compound that is present in various plants, including cumin, a commonly used spice. Several studies have suggested that cuminaldehyde may possess pain-relieving properties by inhibiting the activity of a specific type of pain receptor, known as TRPA1, which plays a critical role in pain transmission [35]. However, there is limited evidence to support the analgesic effects of cuminaldehyde, and further investigations are required to fully comprehend its therapeutic potential as a pain management agent.

Animal studies have shown that cuminaldehyde can reduce pain sensitivity in models of neuropathic pain. The compound has been tested in hot plate, formalin, and acetic acid-induced writhing tests, all of which showed analgesic activity, indicating both central and peripheral effects. Cuminaldehyde’s antinociceptive properties have been demonstrated in these animal models, suggesting that it may have potential as a treatment for pain in humans [17].

The rota rod test was used to evaluate motor activity and provided evidence that the induction of OA was effective. The first evaluation after induction revealed a significant difference between the induced groups, as evidenced by the decreased motor activity of the animals and their impaired gait scores [36]. Due to pain, animals affected by OA experience significantly reduced joint mobility [37]. Treatment with cuminaldehyde showed a significant improvement in motor activity compared to the saline group, suggesting that cuminaldehyde at the tested concentration enhanced the animals’ mobility. The mobility test demonstrated that animals treated with cuminaldehyde exhibited a notable improvement in motor activity from the seventh day of evaluation, which persisted until the final evaluation period (D28) (Figure 1). These findings suggest that cuminaldehyde has potential analgesic activity, resulting in an improvement in the mobility of animals with OA.

Differences in weight distribution between the hind legs are considered to be important indicators of joint discomfort, inflammation, and pain resulting from the induction of osteoarthritis [36]. Weight distribution between the legs is used as an indicator of anti-nociceptive activity, as differences in weight distribution can indicate the presence of joint discomfort and inflammation caused by induced osteoarthritis. The animals treated with cuminaldehyde showed significant improvement in weight distribution on the affected leg compared to the saline group and indomethacin group on days 7, 14, and 28 of the experiment, with over 40% weight distribution on the affected leg. On day 28, the animals treated with cuminaldehyde exhibited weight distribution equal to the healthy animals, while the saline and indomethacin groups still displayed significant differences in weight distribution between the legs.

The Mouse Grimace Scale is a standardized method for quantifying pain in mice based on the observation of facial expressions. The scale consists of specific changes in facial features such as ear and eye position, whisker movement, and overall shape of the face, that are believed to reflect changes in the mouse’s underlying state of pain or discomfort. The Mouse Grimace Scale is used as a tool to assess pain associated with osteoarthritis in mice, as well as to evaluate the efficacy of pain-relieving interventions. This tool is particularly useful in preclinical research, where it is important to assess the effectiveness of new pain-relieving treatments before they are tested in humans.

The MGS is a tool used to evaluate pain in mice with osteoarthritis and allows to quantify pain in mice with osteoarthritis by observing specific changes in their facial expressions [38]. These changes reflect underlying changes in the mouse’s state of pain or discomfort and are scored on a standardized scale [21]. The MGS is particularly useful in preclinical research for evaluating the efficacy of pain-relieving treatments for osteoarthritis in mice. It objectively assesses pain in mice and determines the effectiveness of new treatments before they are tested in humans. The use of the MGS helps to improve the accuracy and reliability of pain assessment in mice with osteoarthritis and ensures that these animals are protected from unnecessary suffering in research [39]. The MGS demonstrates that cuminaldehyde efficiently reduced pain in animals, as their reaction was statistically equivalent to that of animals treated with indomethacin and considerably better than that of animals treated with saline after 14 days of therapy.

The Ahlback Score system is a method of scoring used to assess the severity of osteoarthritis (OA) in the hip joint. This scoring system is based on radiographic changes observed in the hip joint, with higher scores indicating more severe OA. In rat osteoarthritis research, the Ahlback Score system is employed to track the progression of the disease over time. By comparing the scores before and after the induction of osteoarthritis, researchers can evaluate the effectiveness of different treatments in slowing down or reversing the disease’s progression [40]. The validation of the method may depend on the presence of radiological signs of osteoarthritis. In this sub-chronic study, all animal groups displayed joint degeneration, joint space narrowing, bone sclerosis, and osteophytes. Statistical analyses of the Ahlback Score system used to evaluate x-ray images showed a statistically significant difference, indicating that the dose of cuminaldehyde used alters bone remodeling in comparison to the negative control. Additionally, radiographic data of paws affected by osteoarthritis demonstrated that indomethacin and cuminaldehyde produced significant improvements in the degree of joint involvement.

Hyaline cartilage is a critical articular tissue in the development of osteoarthritis. The disease is characterized by subchondral bone sclerosis, which refers to the thickening and increased density of the layer of bone located beneath the cartilage in a joint. In osteoarthritis, subchondral bone becomes thicker and denser due to increased bone formation, resulting in decreased joint flexibility and increased stiffness. These changes can cause pain and limit movement. Moreover, the thickening of the subchondral bone can lead to the formation of bone spurs or osteophytes, which can worsen symptoms further [41]. The histopathological evaluation showed that cuminaldehyde resulted in a significant improvement in the severity or biological progression of the osteoarthritic process compared to saline, indicating the potential efficacy of cuminaldehyde in treating osteoarthritis.

Moreover, the decrease in IFN-γ and IL-6 cytokine levels observed in animals treated with cuminaldehyde suggests a reduction in spinal cord neuroinflammation, which may lead to a decrease in pain signals and an improvement in hypersensitivity and hyperalgesia [42]. IFN-γ is associated with the activation of microglial cells and the sensitization of nerves. On the other hand, IL-6 is a cytokine that promotes the maturation and activation of neutrophils and macrophages, as well as the differentiation and maintenance of cytotoxic T lymphocytes and natural killer cells [43]. In addition, IL-6 also activates astrocytes and microglia in the dorsal spinal cord region [44].

Increased levels of IL-10 in synovial fluid may be associated with lower levels of IL-6 and IFN-γ in the study and may be linked to improved clinical indicators, including a possible reduction in inflammation and an increase in peripheral circulation and nociceptive threshold due to cuminaldehyde treatment. IL-10 is an anti-inflammatory cytokine that suppresses proinflammatory cytokines such as TNF-α, IL-1, and IL-6. It also promotes the proliferation of mast cells and inhibits the production of IFN-γ by natural killer cells [42].

The Real-Time PCR results of *Cuminum cyminum* L. (Apiaceae) essential oil (EO) rich in cuminaldehyde (48%) showed that the EO in significantly inhibition of the mRNA expressions of inducible nitric oxide synthase (iNOS), cyclooxygenase (COX-2), interleukin-1 (IL-1), and IL-6 of RAW 264.7 cells stimulated with lipopolysaccharide (LPS). The western blotting results also demonstrated that the EO blocked LPS-induced transcriptional activation of nuclear factor-kappa B (NF-κB) and inhibited the phosphorylation of extracellular signal-regulated kinase (ERK) and c-Jun N-terminal kinase (JNK), indicating that this cuminaldehyde-rich EO has anti-inflammatory activity [18]. Cuminaldehyde also demonstrated its anti-inflammatory activity by decreasing the serum levels of TNF-α and IL-1β in rats [17] and inhibits 15-LOX [45]. Our results reveal, for the first time, the inhibitory potential of cuminaldehyde on COX enzyme, with a preference for COX-2.

Upon investigating the possible mechanism of the antinociceptive activity of cuminaldehyde, it was discovered that the activity was reversed by naloxone, leading to the conclusion that this molecule functions as an agonist of opioid receptors (OR), particularly the µ type [17]. It is widely recognized that naloxone is a competitive OR antagonist, without agonist action, thus being able to antagonize the adverse effects of opioids or in the treatment of morphine overdose [46]. According to the in silico results, cuminaldehyde was found to have favorable interactions with the three opioid receptors used, which supports the previous experimental literature. In addition, it was observed that cuminaldehyde also has equally favorable interactions with COX-2, which is a novel finding in this study.

Regarding toxicity, the cuminaldehyde-rich *Cuminum cyminum* L. (Apiaceae) essential oil (EO) did not show any in vitro cytotoxic activity against RAW 264.7 cells at concentrations ranging from 0.0005% to 0.01% [18]. When administered at intraperitoneal (i.p.) concentrations ranging from 12.5 to 200 mg/kg, no acute toxicity was observed in animals [17]. Throughout the entire 28-day period of our study, no animal deaths were observed, indicating that prolonged or chronic use of cuminaldehyde does not appear to cause toxicity. This observation is consistent with previous findings in the literature.

## 5. Conclusions

Cuminaldehyde exhibits a multifactorial anti-inflammatory activity, acting through multiple pathways. Its antinociceptive activity occurs via central and peripheral mechanisms and modulates the immune response of the inflammatory process. As a result, cuminaldehyde represents a promising candidate for the development of novel anti-inflammatory and analgesic drugs, given its demonstrated properties and low toxicity to vertebrate organisms. Further research aimed at exploring the full therapeutic potential of cuminaldehyde is warranted.

## Figures and Tables

**Table 1:** Free binding energies obtained by molecular docking of cuminaldehyde and/or drugs with the OpRs of kappa, mu and delta type.

	Target/ΔGbind (kcal/mol)			
Ligand	kappa (κOR) (#6B73)	mu (µOR) (#6DDF)	delta (δOR) (#6PT3)	COX-2 (#1DDX)
Cuminaldehyde	−6.80	−5.60	−6.71	−7.2
Morphine	-	−8.0	-	-
JDTic	−10.70	-	-	-
CVV	−11.80	-	-	-
Naltrindole	-	-	−12.00	-
Indometacin	-	-	-	−8.1

## Data Availability

Not applicable.
